# Green and facile production of high-quality graphene from graphite by the combination of hydroxyl radical and electrical exfoliation

**DOI:** 10.1039/c8ra07880g

**Published:** 2018-12-05

**Authors:** Xin Wang, Long Zhang

**Affiliations:** Jilin Provincial Engineering Laboratory for the Complex Utilization of Petro-resources and Biomass, School of Chemical Engineering, Changchun University of Technology Changchun Jilin 130012 P. R. China zhanglongzhl@163.com +8618686672766; School of Petrochemical Technology, Jilin Institute of Chemical Technology Jilin 132022 P. R. China

## Abstract

A novel, simple and efficient method, involving the combination of hydroxyl radicals and electrical exfoliation of graphite for the green production of high-quality graphene from graphite, was developed for the first time. The process parameters were optimized by single factor experiments; the optimal conditions were 4.0 g graphite dosage, sodium chloride solution with concentration of 5.0% (w/v), applied current strength of 10 mA, and air flow rate of 1.0 L h^−1^ for 3 h. Under the optimized conditions, graphite was successfully exfoliated to graphene. SEM and TEM results revealed that the graphene product has the characteristic features of a thin-layer graphene sheet. XRD results showed that the graphene products still maintained the structure of the carbon atoms or molecules. FT-IR and Raman results indicated that the products have the characteristic peaks and absorption peaks of graphene. AFM imaging results reveal that the layer number of the graphene product obtained by this method is about 3, while the graphene products from the individual hydroxyl radical oxidation and electrical exfoliation processes were 50 and 133 layers, respectively, under the same experimental conditions. The good quality of the graphene product can be attributed to the synergistic effect between the strong oxidation of the hydroxyl radicals and electrical exfoliation. The proposed method has the advantages of simple operation, mild preparation conditions, non-utilization of aggressive reagents, recycling of the reaction medium, *etc.* Thus, this method could serve as a green and efficient alternative for the production of graphene and its derivatives in industry.

## Abstract 2

A novel, simple and efficient method, involving the combination of hydroxyl radicals and electrical exfoliation of graphite for the green production of high-quality graphene from graphite, was developed for the first time.

## Graphical abstract

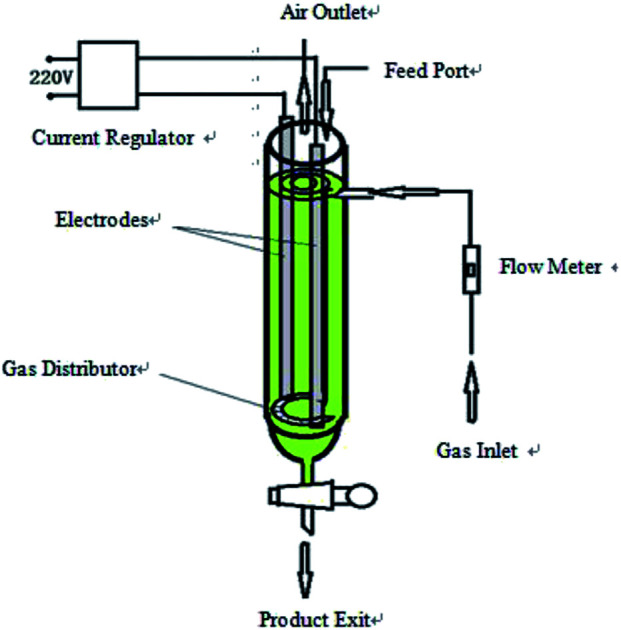

## 1. Introduction

In 2004, graphene was first reported by the British scientists K. S. Novoselov and A. K. Geim *et al.*^1^ Unlike the thicker graphite, graphene is a two-dimensional planar material with a hexagonal lattice-like structure formed by a single-layer of carbon atoms through the sp^2^ hybrid orbital. In graphene, each carbon atom has an unbonded electron, which can move freely at high speed (one-third of the speed of light) in the crystal, so graphene has good electrical conductivity.^1^ At the same time, graphene also has many other excellent properties, such as a large theoretical specific surface area (∼2630 m^2^ g^−1^),^2^ excellent light transmission, (∼97.7%),^3^ and high Young's modulus (∼1.0 TPa).^4^ The special two-dimensional structure of graphene and its various excellent properties endow it with broad application prospects in many fields. Thus far, the most popular methods for the preparation of graphene include micro-mechanical stripping,^5–9^ chemical vapor deposition^10–13^ and oxidation dispersion reduction method.^14–16^ The above preparation methods often use aggressive reagents and are restricted by the relatively high energy consumption, complex operation, environmental pollution, occasionally lower yield and poor product quality. The liquid phase exfoliation method has been extensively studied because it is easy to operate and can obtain higher quality graphene product.^17,18^ However, the use of excess organic solvents often leads to environmental problems and high production expenditure. Therefore, it is necessary to develop a novel green production method to resolve the problems mentioned above.

The hydroxyl radical is the most common and most important of the free radicals. Its oxidation–reduction potential is 2.8 V, which is only lower than that of F.^19^ The hydroxyl radical can react with many organic molecules due to its strong oxidizing capacity. It can initiate and transfer chain reactions and oxidize and decompose organic matter into low toxicity or non-toxic small molecules. The common ways to generate hydroxyl radicals include the Fenton reaction,^19,20^ the Haber–Weiss reaction^21^ and electrochemical methods.^22–24^ These hydroxyl radical generation methods have the disadvantages of high energy consumption and complex production procedures; hence, they are not suitable for mass applications. At present, the hydroxyl radical is mainly used in the field of sewage treatment, sterilization and preservation.^20–23^ Wei *et al.*^24^ have used ˙OH produced by the Fenton reagent to achieve good chemical modification of carbon nanotubes. Feng *et al.*^25^ have introduced ˙OH (produced by UV light irradiation) into the synthesis process for zeolites, and it was found that ˙OH can significantly accelerates the nucleation of zeolite, thus accelerating its crystallization process. However, there have been no reports on the application of hydroxyl radicals for the exfoliation of graphite to prepare graphene. Therefore, in the present study, for the first time, we used hydroxyl radical exfoliation for the facile and green production of high-quality graphene from graphite.

To achieve these goals, we designed a new apparatus for the production of ˙OH and exfoliation of graphite. The effect of the process parameters (such as exfoliation time, sodium chloride solution concentration, graphite dosage, applied current strength and air flow rate) on the production of graphene were investigated systematically. Hydroxyl radical oxidation and electrical exfoliation were also performed individually for comparison. The experimental results show that graphite has been successfully exfoliated into graphene by this method. The layer number of graphene product was determined to be about 3, while that of the graphene product from the individually performed hydroxyl radical oxidation experiment and electrical exfoliation experiment were 50 and 133, respectively, under the same experimental conditions.

## 2. Experimental

### 2.1 Materials and instruments

Flake graphite (0.5 mm) was purchased from Sinopharm Chemical reagent Co. (Shanghai, China). Sodium chloride (AR) and ethanol (AR) were purchased from Sinopharm Chemical reagent Co. (Shanghai, China).

**Fig. 1:**
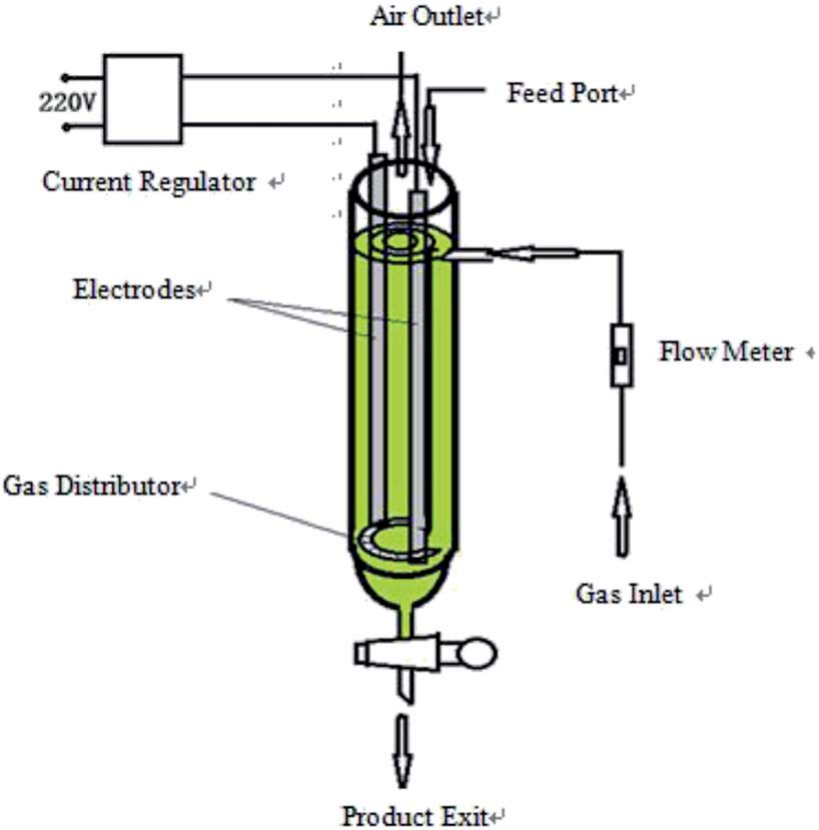
Diagram of the hydroxyl radical generation and graphite exfoliation apparatus.

The hydroxyl radical production and graphite exfoliation apparatus was designed and manufactured by our laboratory; the detailed device diagram was shown in Fig. 1. The analytical balance (TG328A) was purchased from Balance instrument factory (Shanghai, China). The pumping equipment was purchased from Guohua Electric Co. (Shanghai, China). The vacuum drying oven was purchased from Anteing Electronic Instrument Factory (Shanghai, China).

### 2.2 Experimental devices

The device is a high efficiency hydroxyl radical generation apparatus. The apparatus consists of a current regulator, electrode (graphite or stainless steel) system, gas inlet and distributor, flow meter, feed port, air outlet and product exit. The reaction liquid (water and electrolyte) and graphite were added from the feed port. The air flow rate was adjusted by a flow meter. The air and graphite were evenly distributed in the reaction liquid under the effect of an air distributor; the intensity of the applied current was adjusted by the current regulator. When an electric current was applied, water decomposed and oxidized to produce ˙OH in the reactor with graphite as the catalyst, which subsequently reacted with the graphite to produce graphene.

After completion of exfoliation, the obtained graphene was discharged out the reactor from the product exit, filtered and dried to get the product. The diagram of the apparatus is shown in Fig. 1.

### 2.3 Preparation procedures

Graphite powders were loaded in the hydroxyl radical apparatus containing a volume of sodium chloride solution according to the experimental design. The applied current strength in the process was adjusted by a variable resistor having 50–500 Ω. The exfoliation experiments were started when the electric current was applied, water was decomposed and oxidized to produce ˙OH in the reactor with graphite as the catalyst, which subsequently reacted with graphite to produce graphene. The flake graphite added to the reaction liquid also served as a catalyst. This resulted in the simultaneous generation of the hydroxyl radical and the reaction between the hydroxyl radical and the graphite in solution. After the designated exfoliation time, the solution was filtered, and the solid was dried and ground to powder for characterization, and the filtrate was reused for the next experimental run.

We also investigated the effects of different preparation parameters on the quality of the graphene products. Five process factors (exfoliation time, sodium chloride solution concentration, graphite dosage, applied current strength and air flow rate) were defined and adjusted in the exfoliation process.

### 2.4 Characterization

#### 2.4.1 Morphological elucidation

Morphological information on the samples was obtained using a SU8020 Hitachi scanning electron microscope and H800 transmission electron microscope (Tokyo, Japan). Further morphological information was obtained by atomic force microscope.

#### 2.4.2 Structural investigation

The molecular structure of the graphene product obtained was identified by X-ray diffraction. After vacuum drying and grinding to powders, the samples were scanned and recorded using an X-ray diffractometer (Rigaku, Japan) with an X-ray generator from 15° to 60° of 2*θ* (Bragg angle), using Cu/Kα irradiation at 55 mA and 60 kV. Structural information of the product was obtained by Raman (Horiba JY) and FT-IR spectroscopy. Fourier transform infrared spectra (FT-IR) were obtained with an IS50 FT-IR system spectrometer (Horiba JY). The wave number range scanned was 4000–400 cm^−1^. After vacuum drying and grinding to powders, the samples and KBr were compacted into disks and analyzed.

## 3. Results and discussion

### 3.1 Optimization of process parameters

#### 3.1.1 Effect of reaction time

**Fig. 2:**
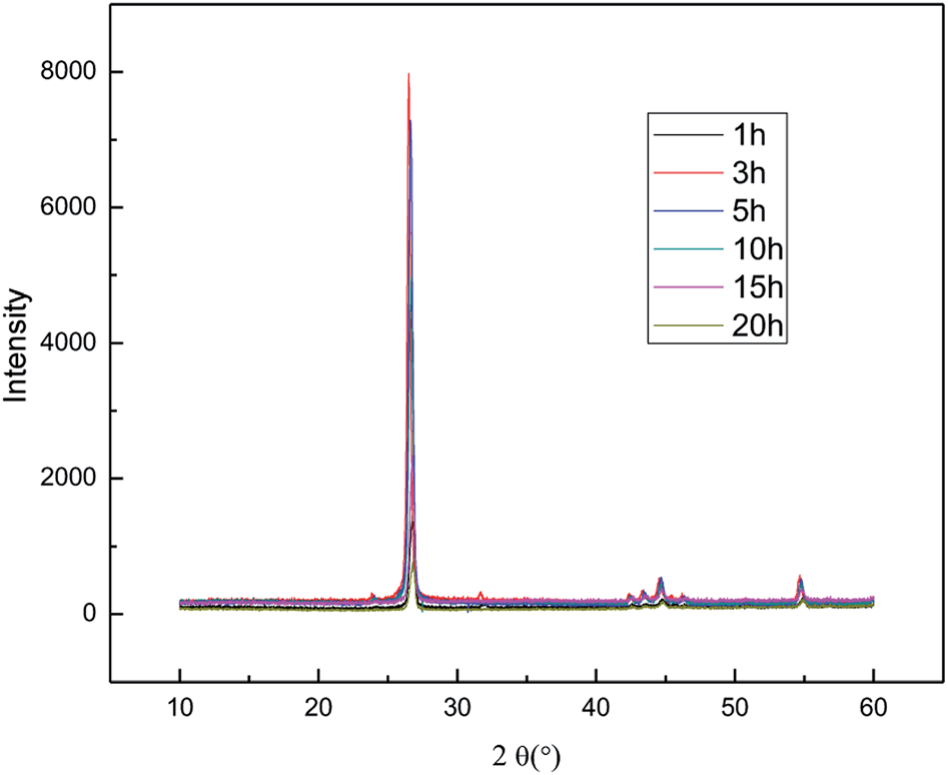
XRD patterns of products obtained from different reaction times.

**Fig. 3:**
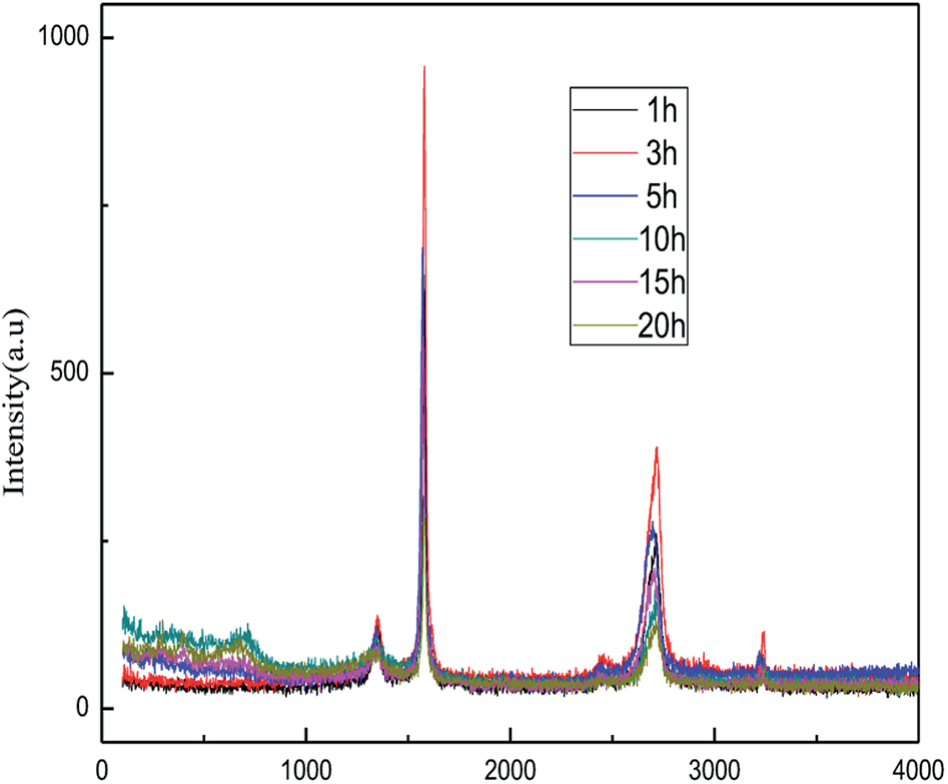
Raman spectra of products obtained from different reaction times.

**Fig. 4:**
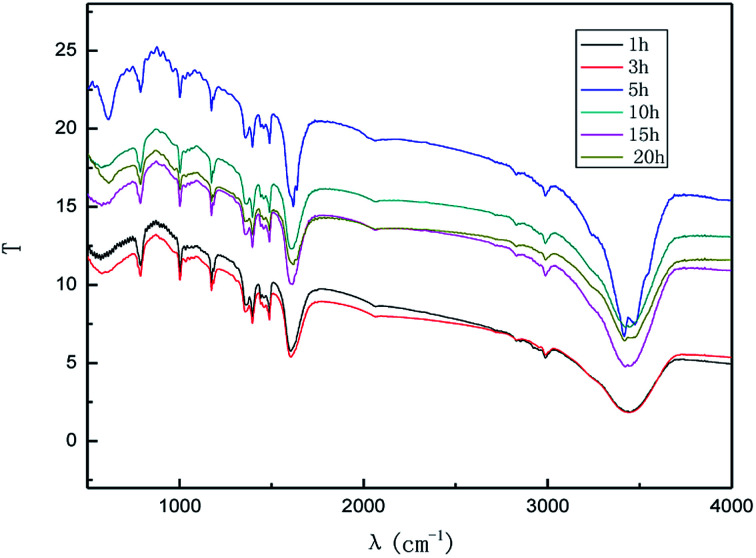
FT-IR patterns of products obtained from different reaction times.

Fig. 2 shows the XRD patterns of the products obtained at different reaction times (1–20 h). The experiments were set as follows: the concentration of sodium chloride solution was 5% (w/v), the air flow rate was 1.0 L h^−1^, the applied current strength was 15 mA, and the dosage of the graphite powder was 2.0 g. In Fig. 2, it is demonstrated that the diffraction angle of the XRD characteristic peak for the product is basically the same as that of the natural flake graphite, indicating that the product has the same carbon-based material composition and the same internal carbon atoms or molecular structural form as the natural flake graphite.^26–28^ Visibly, the position of the XRD characteristic peaks for the products obtained at different exfoliation times remains the same, indicating that time does not change the internal structure of the product. The Raman spectra of the products are shown in Fig. 3. Fig. 4 shows the FT-IR results for different exfoliation times. Fig. 3 and 4 show that as the time was extended, the intensity of the characteristic peak of graphene increased initially and then decreased, with a maximum reached at 3 h. This revealed that the oxidation of the hydroxyl radical and the electrical exfoliation show a synergic effect. In the beginning, more hydroxyl radicals were produced with the increase in time, which strengthened the synergistic effect. However, graphene aggregation was more prominent when the time was extended. This resulted in the decrease in the intensity of the characteristic peak. Thus, the appropriate reaction time was set as 3 h, which was adopted in the subsequent experiments.

#### 3.1.2 Effect of sodium chloride solution concentration

**Fig. 5:**
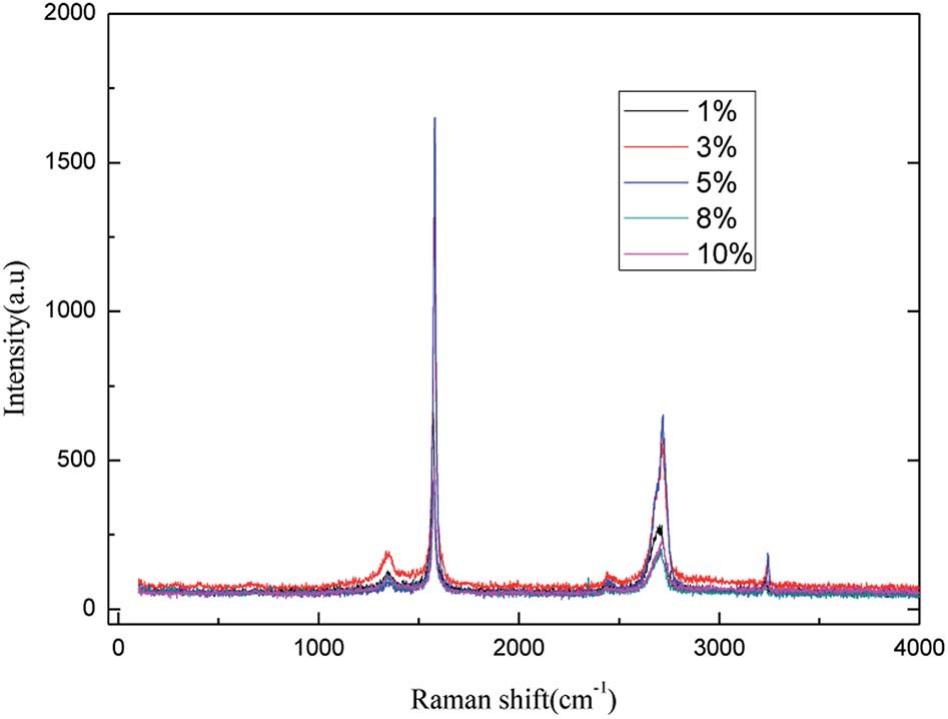
Raman spectra of products obtained with various electrolyte concentrations.

**Fig. 6:**
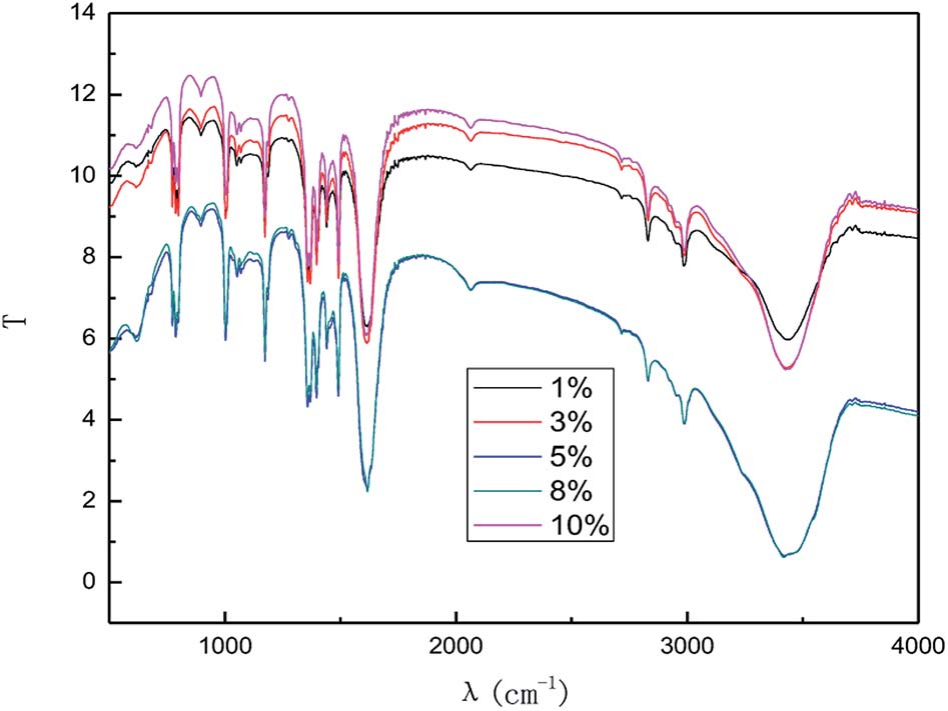
FT-IR patterns of products obtained with various electrolyte concentrations.

The effect of the electrolyte concentration on the preparation of the graphene was investigated for concentrations from 1.0% (w/v) to 10% (w/v); the air flow rate was 1.0 L h^−1^, the applied current strength was 15 mA, and the dosage of graphite powder was 2.0 g. The results are shown in Fig. 5 and 6.

It can be easily seen from Fig. 5 that the intensity of the characteristic peak for graphene increased with the increase in electrolyte concentration up to a maximum at 5% (w/v), and then decreased. This phenomenon can be attributed to the increase in conductivity of the solution when a higher electrolyte concentration is used. Moreover, good conductivity leads to good ˙OH formation and electrical exfoliation effect. Furthermore, the hydroxyl radicals generated from water decrease due to less water at higher electrolyte concentration, which leads to the intensity of the characteristic peak of graphene to decrease. Hence, when the electrolyte concentration is less than 5.0% (w/v), the increase in the conductivity dominated, and when the concentration exceeds 5.0% (w/v), the decrease in hydroxyl radical content was more dominant. Literature ^29–33^ reports also indicate the optimal electrolyte concentration for the production of hydroxyl radicals in different electrolyte systems using different production methods. Li *et al.*^31^ used a highly efficient generation reactor to produce hydroxyl radicals, and showed that the optimum electrolyte concentration was in the range of 3.0–6.0% (w/v). Therefore, it is reasonable to expect that an optimal concentration of electrolyte may exist. Moreover, the higher concentration of sodium chloride solution results in stronger corrosion of the electrodes. As can be inferred from the results, 5.0% (w/v) electrolyte concentration was found to be optimal for the investigation.

#### 3.1.3 Effect of graphite dosage

**Fig. 7:**
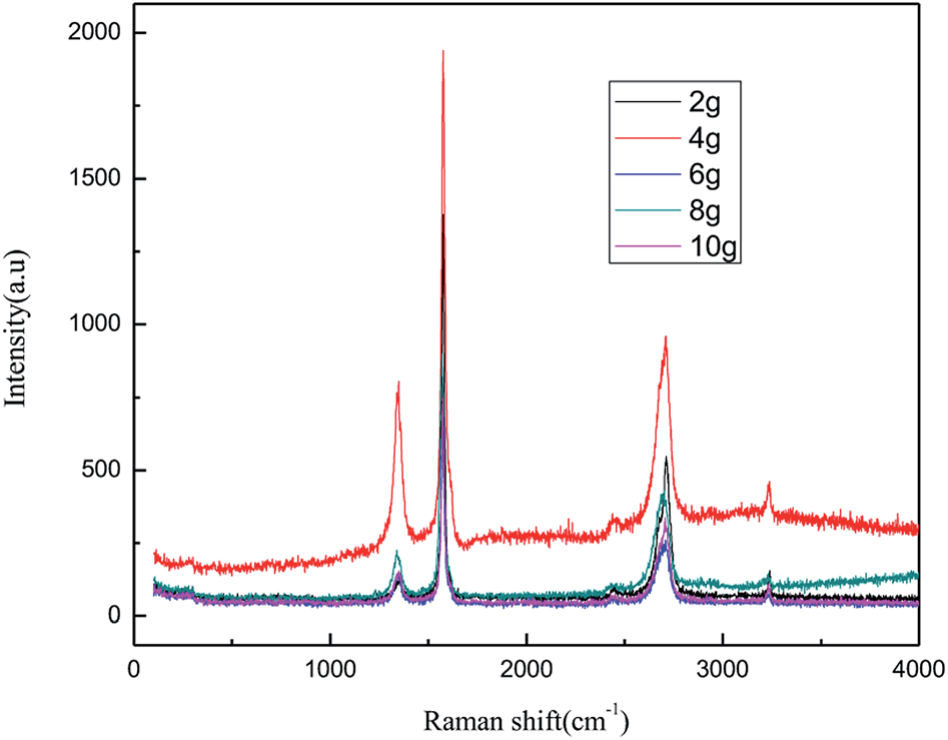
Raman spectra of products obtained from different graphite dosage.

**Fig. 8:**
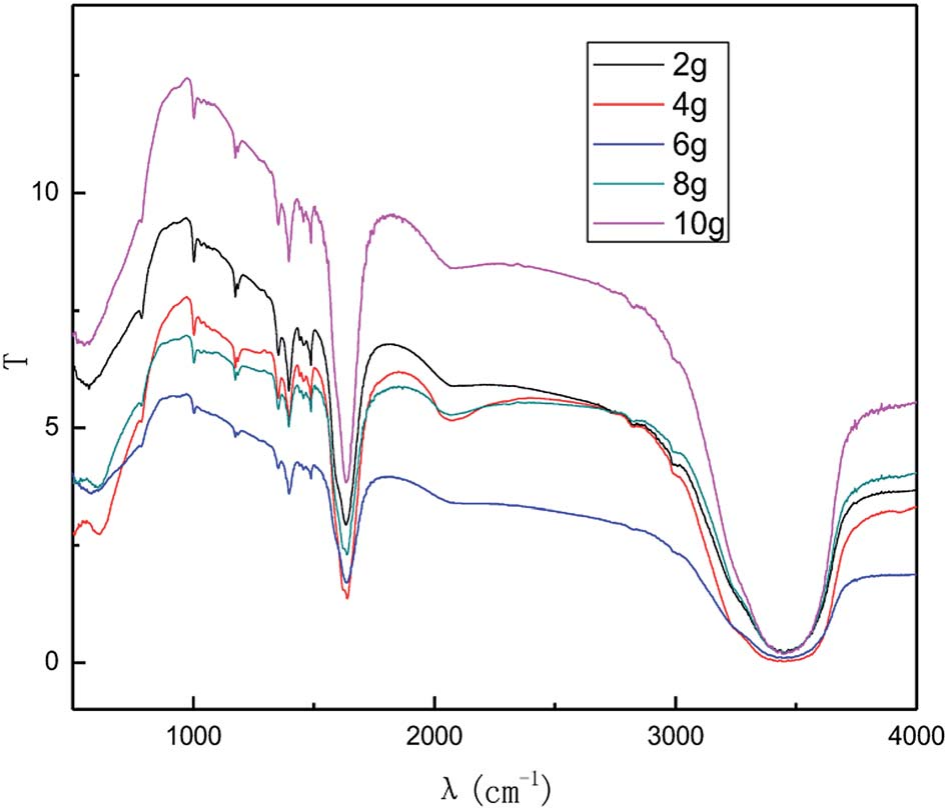
FT-IR patterns of products obtained from different graphite dosage.

The dosage of graphite is a key parameter because graphite is not only a raw material for the reaction, but also has catalytic activity for the process. Fig. 7 and 8 show the effect of the graphite dosage on the exfoliation of graphene at the air flow rate of 1.0 L h^−1^, applied current strength of 15 mA for the production process, and dosage of graphite powder varied from 2.0 g to 10.0 g.

As seen from Fig. 7 and 8, the intensity of the characteristic peak of graphene first increased with the increase in graphite dosage, achieving a maximum at 4.0 g, and then decreased. This is because higher dosage of graphite leads to better catalytic activity, which improved the intensity of the characteristic peak of graphene. However, higher graphite dosage induced more graphene aggregation and uneven distribution, resulting in the decline in the intensity of the characteristic peak of graphene. It can be seen from the results that a suitable graphite dosage is 4.0 g.

#### 3.1.4 Effect of applied current strength

**Fig. 9:**
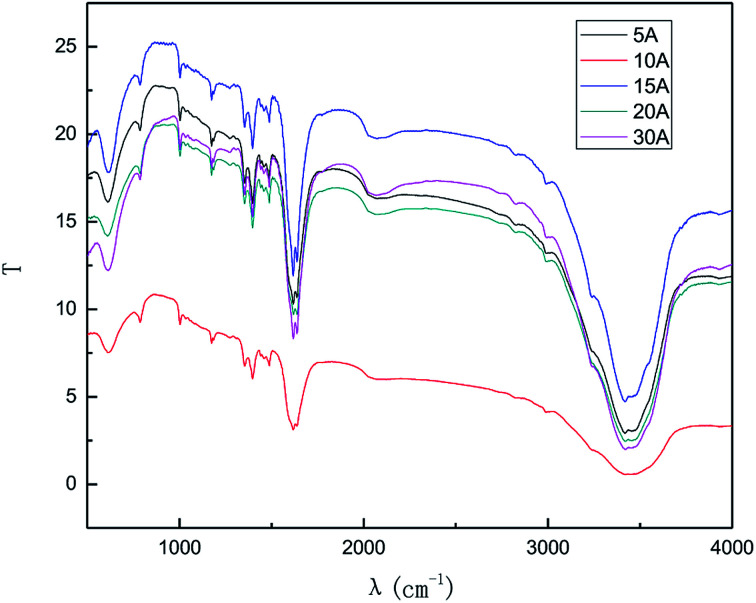
Raman spectra of products obtained with different direct current strengths.

**Fig. 10:**
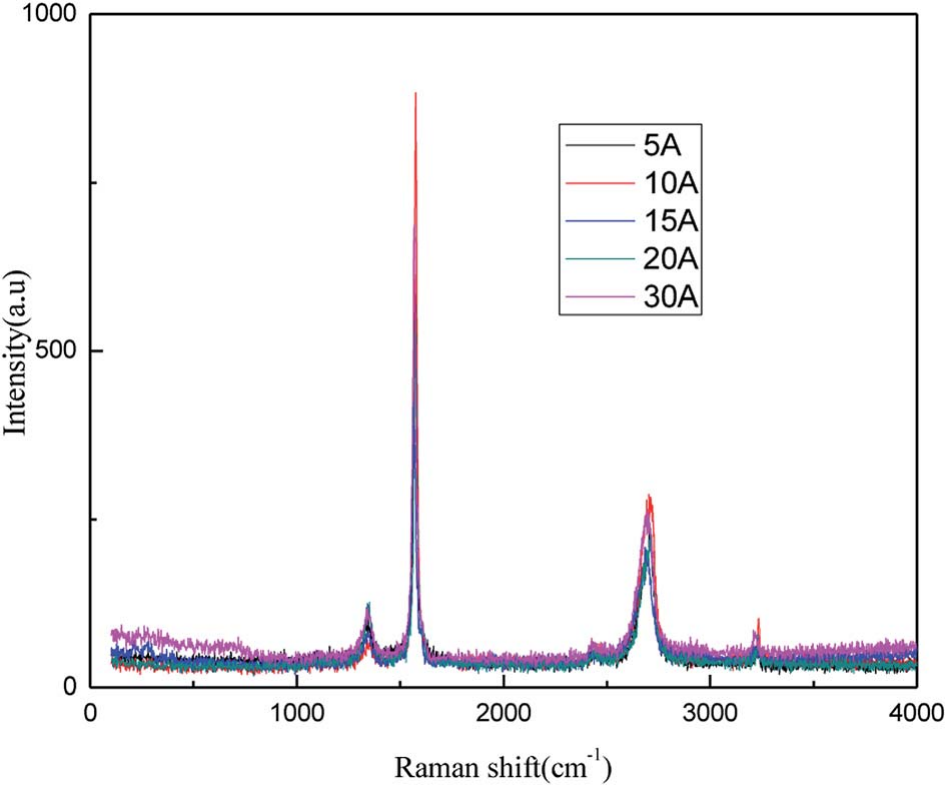
FT-IR patterns of products obtained with different direct current strengths.

The effects of the applied current strength on the exfoliation process were investigated at applied current strengths ranging between 5 mA and 30 mA. The results are shown in Fig. 9 and 10.

The results from Fig. 9 and 10 indicate that the intensity of the characteristic peak of graphene initially enhanced with the increase in the applied current strength, reaching a maximum at 10 mA, and then decreased. This may be attributed to the combined effects of hydroxyl radical exfoliation and electrical exfoliation. Over a certain range, a relatively higher applied current strength, indicating higher power energy input, can result in higher hydroxyl radical production and better electrical exfoliation effect. However, when the current is too high, the cathode and anode will exhibit the side effects of hydrogen and oxygen precipitation.^34–36^ The reactions are shown below ((1) and (2)). The bipolar side effects lead to a decrease in hydroxyl radical production, current efficiency and the effect of electrical exfoliation.

1 2H_2_O − 4e → O_2_↑ + 4H^+^

2 2H^+^ + 2e → H_2_↑

In order to ensure an improved preparation process, 10 mA was selected as the optimal applied current strength.

#### 3.1.5 Effect of the air flow rate

**Fig. 11:**
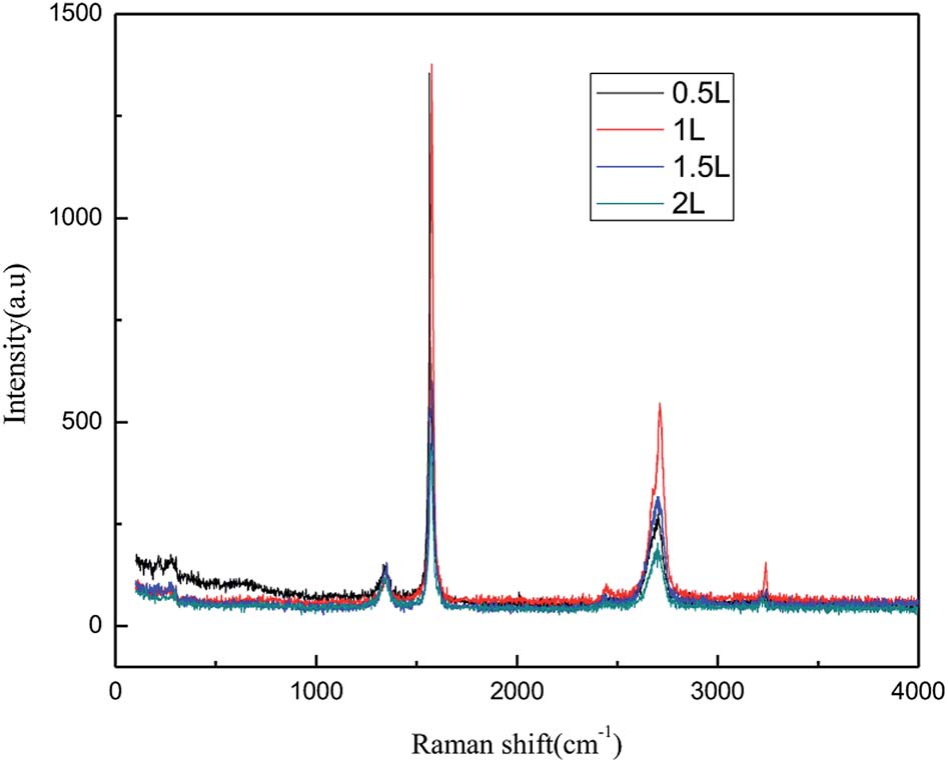
Raman spectra of products obtained with various air flow rates.

**Fig. 12:**
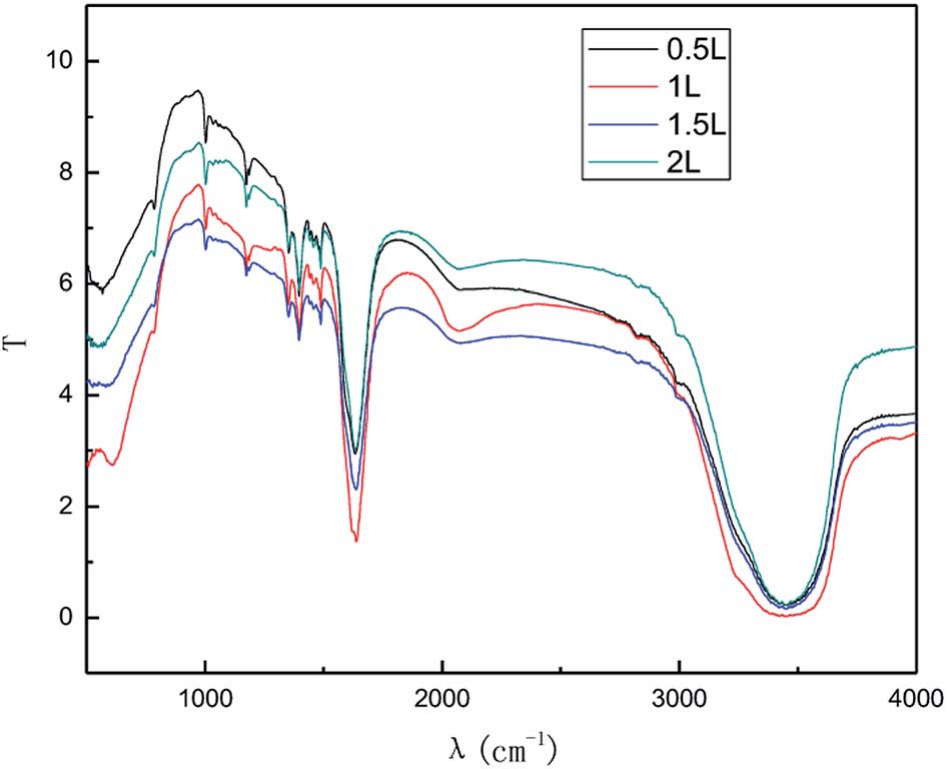
FT-IR patterns of products obtained with various air flow rates.

In general, a higher air flow rate can result in a more even dispersion of graphite in solution and favor better contact of graphite with the produced ˙OH, leading to an improvement in the preparation. From the economic perspective, employing a high air flow rate is not considered as cost-effective due to the higher operating cost and energy consumption. The effect of the air flow rate on the exfoliation of graphene is shown in Fig. 11 and 12. The experiments were performed in the abovementioned optimal conditions. In Fig. 11 and 12, it can be seen that the intensity of the characteristic peak of graphene first increased with the increase in air flow rate, up to a maximum at 1.0 L h^−1^, and then decreased. This is because the higher air flow rate can increase the mass transfer rate and ˙OH formation initially. However, when the air flow rate became too high, the mass transfer interfacial area decreased at unit time, which was not favorable for ˙OH formation and the mass transfer rate. This led to the diminishing of the characteristic peak of graphene. Hence, the air flow rate of 1.0 L h^−1^ was selected.

In summary, the optimal exfoliation conditions for graphene were as follows: electrolyte concentration, 5.0% (w/v); graphite dosage. 4.0 g; exfoliation time, 3 h; applied current strength, 10 mA; air flow rate 1.0 L h^−1^. At these conditions, the layer number of the graphene was 3.

### 3.2 Mechanism discussion

#### 3.2.1 Scanning electron microscopy and transmission electron microscopy (SEM, TEM) analysis

**Fig. 13:**
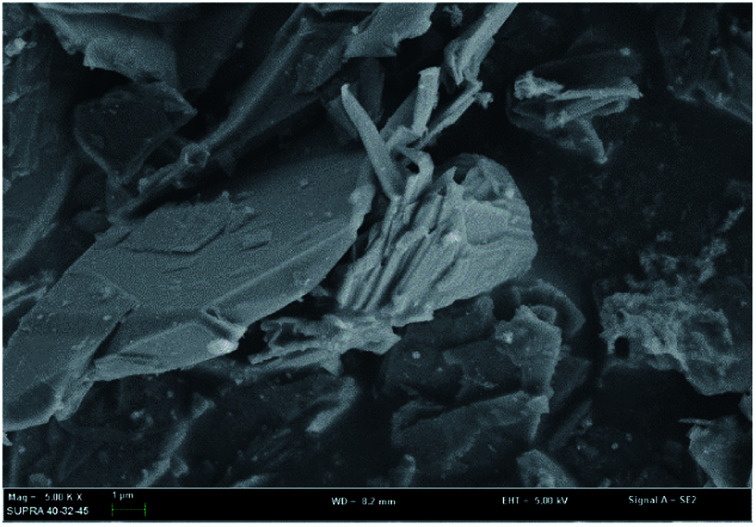
SEM image of samples produced under the optimum conditions.

**Fig. 14:**
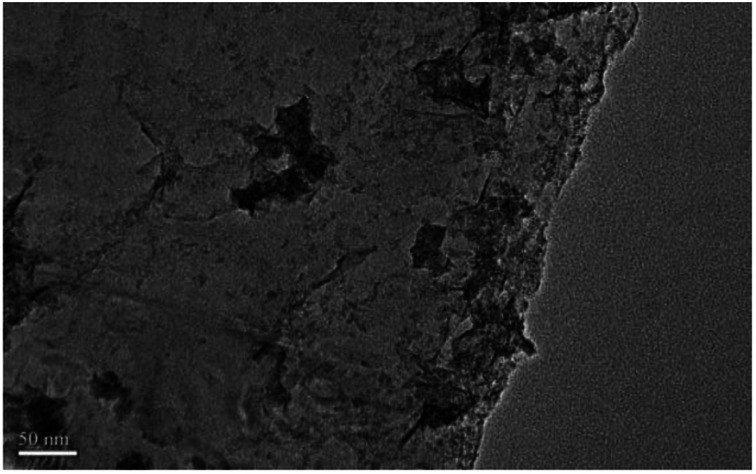
TEM image of samples produced under the optimum conditions.

In order to investigate the mechanism of this process, scanning electron microscopy was used to observe the morphological variation of the graphene products obtained at optimum exfoliation conditions. It can be seen from Fig. 13 that the surface of the graphite has cracks and ravines after being treated in our procedure. In order to further observe the structural changes of the products, transmission electron microscopy was performed. The results are shown in Fig. 14. It can be seen from Fig. 14 that the presence of wrinkles and folds on the sheet is a characteristic feature of the thin-layer graphene sheets,^37^ which indicates that graphite is successfully exfoliated into a uniform thin layer of graphene.

#### 3.2.2 X-ray diffraction (XRD) analysis

**Fig. 15:**
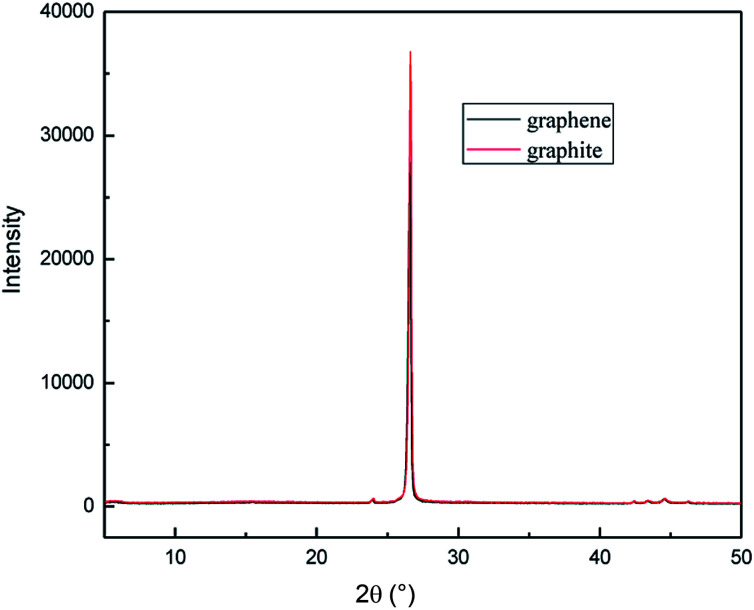
XRD patterns of raw materials and graphene product.

To investigate the crystal structures of the graphene product obtained by this method, the XRD patterns were measured for the samples. Fig. 15 shows the wide-angle XRD patterns of the two samples. It can be seen that the graphite had carbon atoms or molecular structure with a diffraction peak at the 2*θ* angle of about 27°, which was assigned to the (002) plane.^38^ The XRD pattern of the graphene product has almost the same diffraction peak as graphite, which indicates that the obtained graphene maintains the structure of the carbon atoms or molecules; however, the diffraction peak was broadened with lower intensity. This was due to the narrower layer when graphite transformed to graphene.^39^ The degree of disorder in the crystal structure increases with the reduction in the integrity. The results of XRD analysis are in good agreement with that of the TEM.

#### 3.2.3 Fourier transform infrared spectra (FT-IR) analysis

As a relatively easy method, FT-IR spectroscopy has been widely used in graphene research, from which the direct structural information and changes due to various chemical treatments can be obtained.

**Fig. 16:**
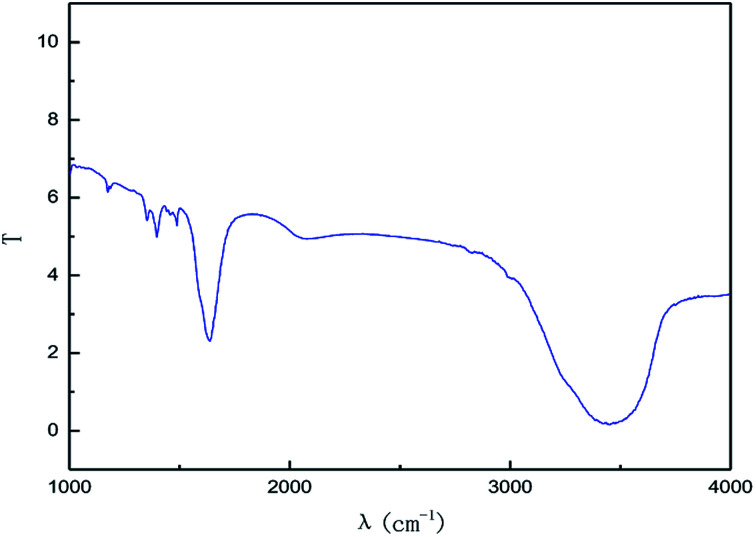
FT-IR pattern of graphene product.

The effect of the hydroxyl radical on the graphene structure was studied by FT-IR spectroscopy. The results are shown in Fig. 16. It can be seen that the main absorption peaks were at 1045 cm^−1^, 1264 cm^−1^, 1512 cm^−1^, 1620 cm^−1^ and 3400 cm^−1^. The peak at 1045 cm^−1^ was caused by the C–OH vibration.^42^ The peaks at about 1264 cm^−1^ and 1512 cm^−1^ were caused by the C–O–C vibration^39,43^ and the stretching of C–O bond, respectively.^44,45^ The absorption peak at 1620 cm^−1^ is attributed to the sp^2^ structure of the graphite crystal C=C stretching vibration peak.^40^ The broad and strong absorption peak in the fingerprint region at around 3000–3700 cm^−1^ is attributed to the OH stretching vibration peaks.^39,41,42^ These results further indicate that the preparation method does not change the carbon-based structure and introduces hydroxyl radicals into graphene at the same time. The FT-IR spectroscopy results are in good agreement with those of XRD and TEM.

#### 3.2.4 Raman spectroscopy (Raman) analysis

**Fig. 17:**
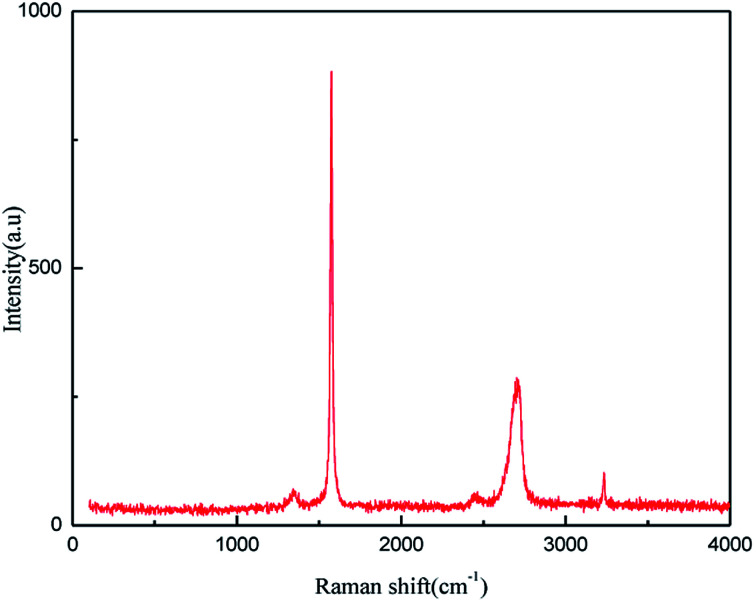
Raman pattern of graphene product.

The application of Raman spectroscopy allows graphene to be accurately characterized. The Raman spectroscopy results for the product produced under the optimum exfoliation conditions are shown in Fig. 17. As shown in Fig. 17, the Raman peaks at l351 cm^−1^, l582 cm^−1^, 2720 cm^−1^ and 3250 cm^−1^ are the characteristic graphene peaks, which can be attributed to the D, G, D′ (2D) and G′ modes, respectively. The G and D peaks evolve due to the sp^2^ structure. The G peak evolves due to the stretching motion of all the sp^2^ atom pairs in the carbon ring or the long chain.^46,47^ The D peak is generated by the sp^2^ respiratory vibration mode in the carbon ring, which indicates that some sp^2^ hybridized carbon atoms in the structure are transformed into sp^3^ hybrid structures. This transformation may be caused by the cleavage of the C=C double bonds in the graphite layer. In addition, the intensity ratio of the G band to the D band also represents the sp^2^/sp^3^ carbon atom ratio.^47,48^ As can be seen from Fig. 17, the intensity of the G band is far stronger than that of the D band, indicating that the carbon skeleton structure has not changed, which is in good agreement with the FT-IR spectral analysis. The D′ (2D) and G′ modes belong to the sum and frequency of the disordered Raman modes and are Raman allowed in the presence of intact graphite crystals and defects; hence, they have strong Raman signals. Graphene has a low degree of graphitization. Therefore, the D′ (2D) and G′ modes are usually very weak and wide. The second-order Raman peak is not considered here.^42^ The graphene absorption peak at 2720 cm^−1^ moves slightly at different layers. Femri *et al.*^49^ studied the change in the 2D peak position with the number of layers of graphene and used the double resonance model to explain this phenomenon. In order to further study the number of layers in the graphene products, AFM test was performed.

#### 3.2.5 Atomic force microscope (AFM)

**Fig. 18:**
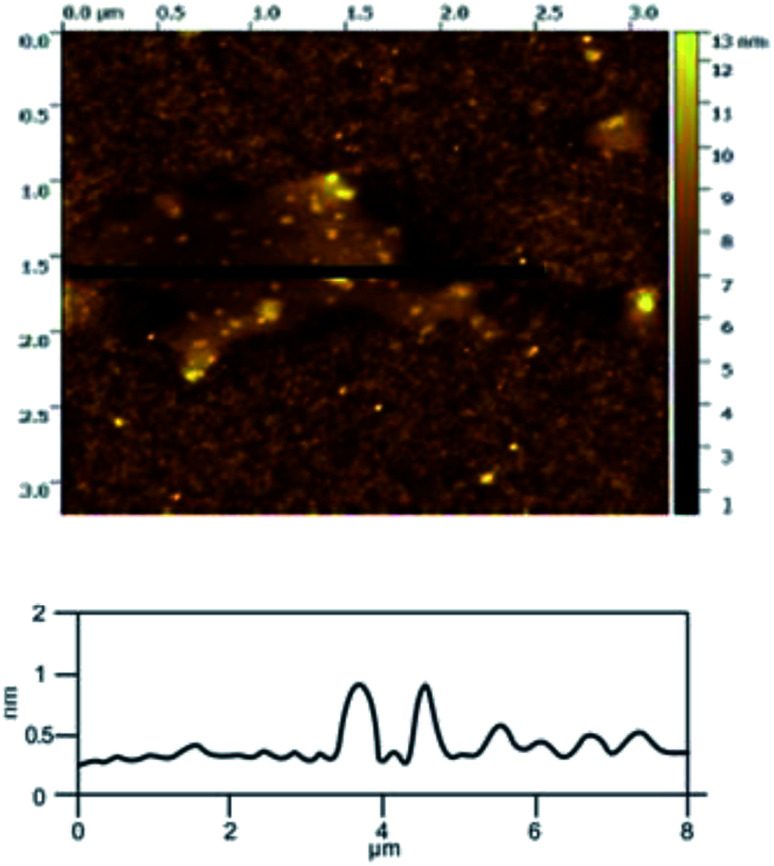
AFM image of the sample produced at under optimum conditions.

The use of AFM makes it possible to observe single-layer graphene. The thickness of graphene is only 0.335 nm, which makes it hard to observe by SEM, but it can be clearly observed in AFM. Atomic force microscopy is the most direct and effective method to characterize graphene materials. Geim *et al.*^5^ found that single layer graphene attached to an Si wafer with a certain SiO_2_ layer thickness (300 nm) covering the surface can be clearly observed under the atomic force microscope. The number of layers in the graphene product (produced under the optimal exfoliation conditions) was analyzed by AFM. From the results shown in Fig. 18, it can be seen that the thickness of the graphene product is 1.0 nm, which indicates that the number of layers in the graphene product is about 3. The AFM results indicate that the quality of the graphene is good.

#### 3.2.6 Exfoliation mechanism

**Fig. 19:**
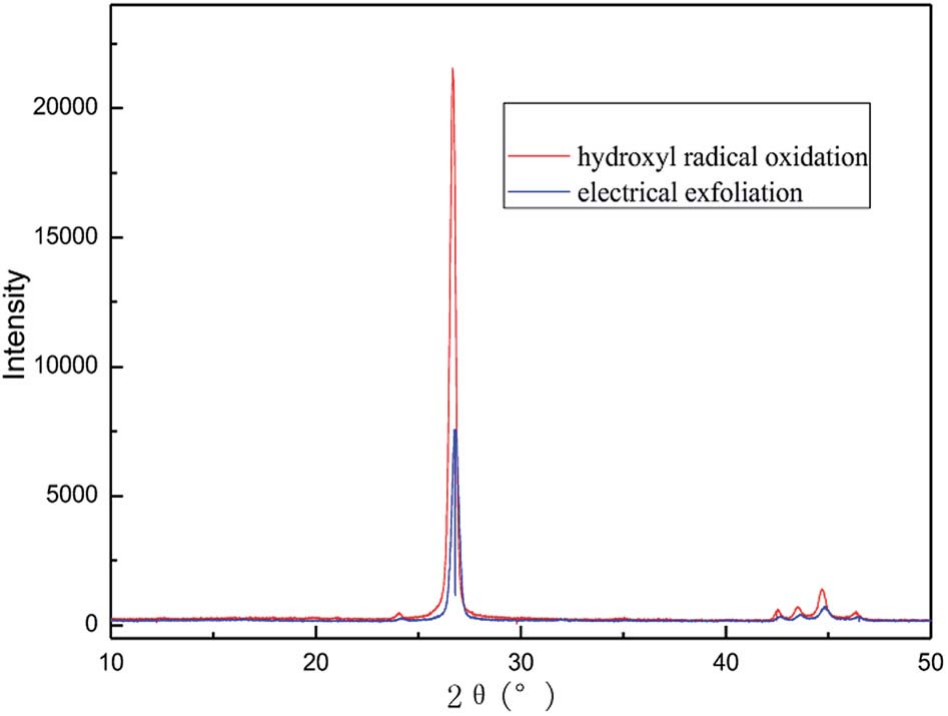
XRD patterns of samples obtained by hydroxyl radical oxidation and electrical exfoliation experiments, respectively.

**Fig. 20:**
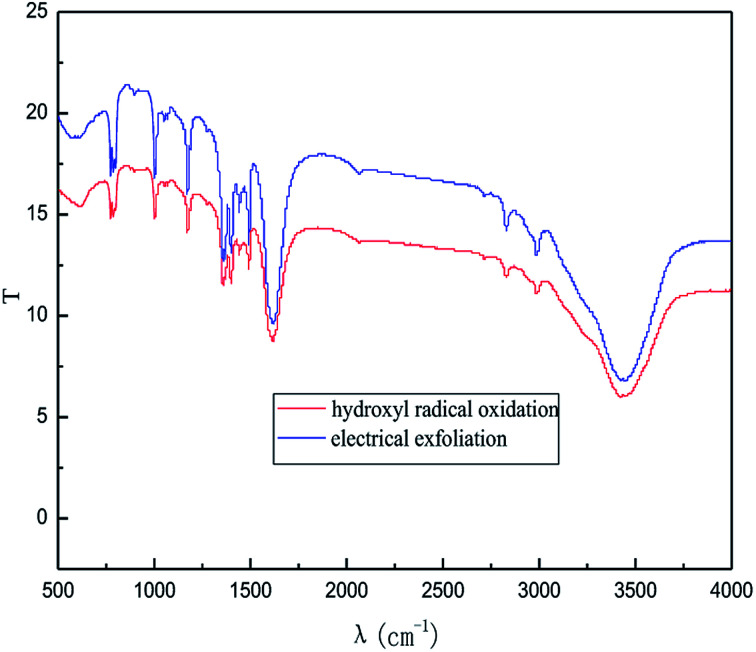
FI-IR patterns of samples obtained by hydroxyl radical oxidation and electrical exfoliation experiments, respectively.

**Fig. 21:**
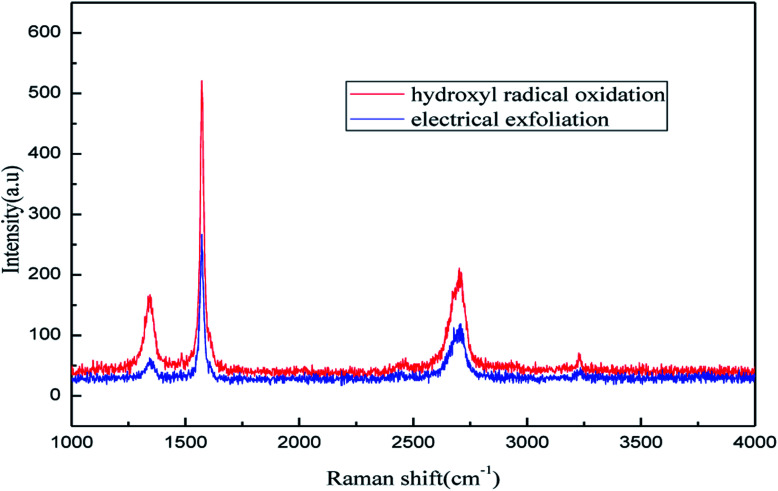
Raman patterns of samples obtained by hydroxyl radical oxidation and electrical exfoliation experiments, respectively.

**Fig. 22:**
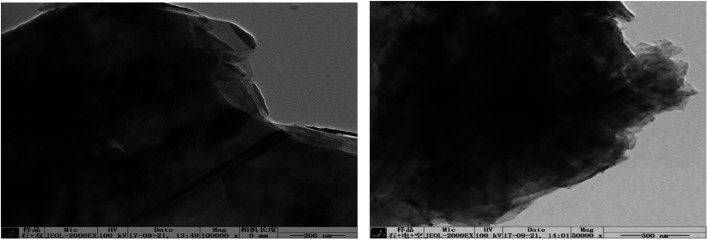
TEM images of samples obtained by hydroxyl radical oxidation and electrical exfoliation experiments, respectively.

**Fig. 23:**
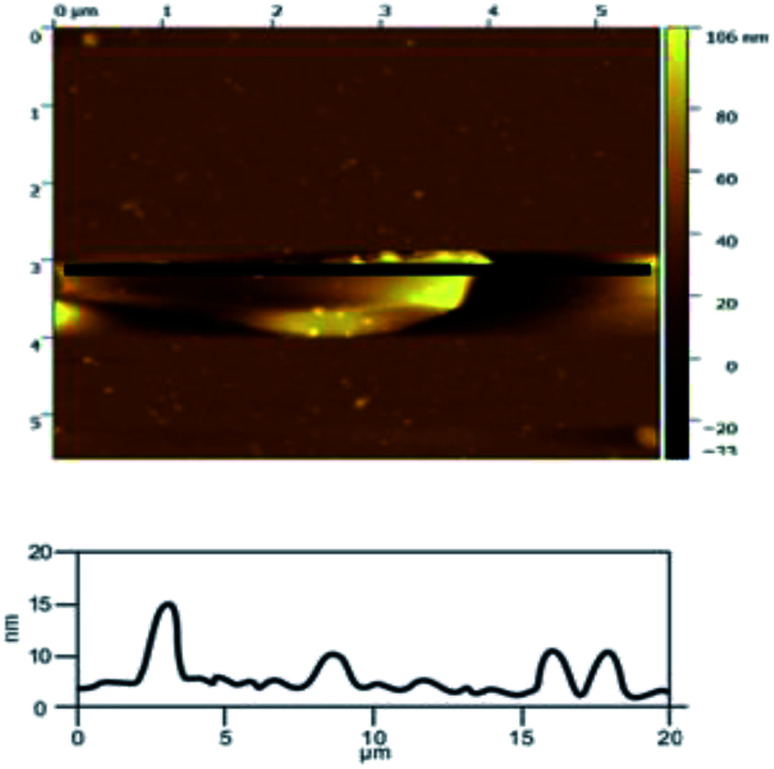
AFM image of sample obtained by hydroxyl radical oxidation exfoliation.

**Fig. 24:**
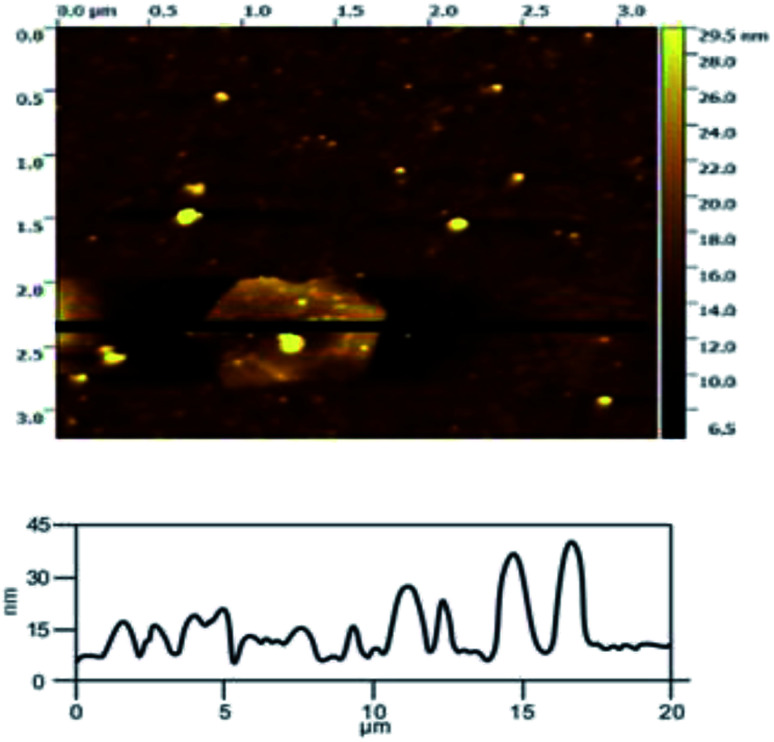
AFM image of sample obtained by electrical exfoliation.

In order to illustrate the effect of the hydroxyl radicals and electrical exfoliation on the exfoliation of graphite to produce graphene, the hydroxyl radical oxidation exfoliation experiments and electrical exfoliation experiments were performed individually. The hydroxyl radical oxidation exfoliation experiment was performed with the concentration of hydrogen peroxide at 5.0% (for the generation of hydroxyl radicals), graphite dosage of 4.0 g and reaction time of 3 h. The electrical exfoliation experiment was performed at the current of 10 mA, graphite dosage of 4.0 g and reaction time of 3 h. The results are shown in Fig. 19–22. From Fig. 19, we can see that the 2*θ* angle diffraction peak for the products obtained by the hydroxyl radical oxidation and electrical exfoliation experiment were all the same for our graphene products, which indicates that both methods can produce graphene. Fig. 20 and 21 show the FT-IR and Raman results for the hydroxyl radical oxidation and electrical exfoliation products. It can be seen that the main absorption peaks are the same as the graphene products, in agreement with the XRD results. However, the intensity of the absorption peaks in the XRD patterns of the graphene products obtained from hydroxyl radical oxidation experiment and electrical exfoliation experiment are much weaker than that of the graphene product obtained by our method, which may prove that the action mechanism of our method is the synergistic effect between the hydroxyl radical oxidation and the electrical exfoliation. The TEM images in Fig. 22 show that the samples obtained by the hydroxyl radical oxidation experiment and electrical exfoliation experiments are not of good quality compared with that produced by our method. In order to further identify the number of layers of graphene in the products produced using the different exfoliation methods, AFM test was performed. The results are shown in Fig. 23 and 24. From Fig. 23 and 24, it can be seen that the thickness of the graphene products are 15 nm and 40 nm, respectively, which indicates that the number of layers of graphene in the products is 50 and 133, respectively. Fig. 18 shows that the number of layers of graphene product obtained by our method is about 3. This result proves that our green production method showed good exfoliation effects.

#### 3.2.7 X-ray photoelectron spectrometer (XPS)

**Fig. 25:**
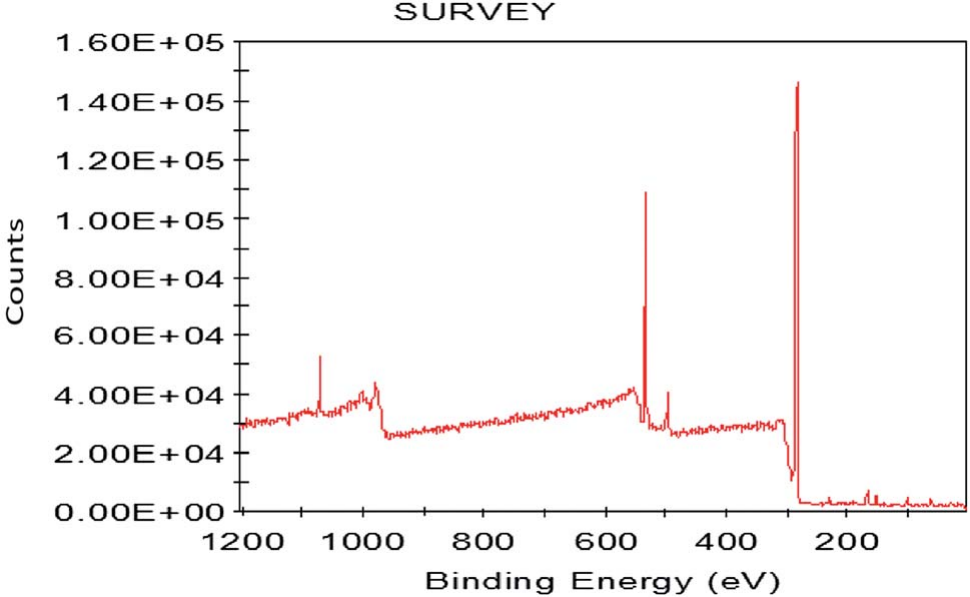
XPS patterns of graphene product.

To further analyze the elements of the graphene product, we performed the XPS test. The experimental results are shown in Fig. 25. As shown in Fig. 25, the binding energy at 282.55 eV and 530.33 eV are characteristic graphene peaks, which are attributed to C 1s and O 1s. The C 1s peak is mainly observed due to the carbon structure of the graphene and the O 1s is mainly observed due to the hydroxyl radicals. Further quantitative calculations found that the carbon element content of the graphene product was 82.18% and the oxygen element content was 17.82%. The experimental results show that our preparation method produces good graphene products with no impurities. The XPS results are in good agreement with those of FT-IR and XRD analysis.

### 3.3 Yield of the production

The graphene dispersion has good Lambert–Beer behavior, and the concentration of the graphene dispersion has a good linear relationship with the ultraviolet absorbance. *A*/*I* = *αC*, where *A*/*I* is the absorbance value of the unit cuvette length, *C* is the concentration of the graphene dispersion, and *α* is the extinction coefficient of the graphene dispersion. It is reported in the literature^50^ that the extinction coefficient (*α*) of the graphene dispersion at *λ* = 660 nm is 3620 L^−1^ g^−1^ m^−1^.

The experiment was performed under the previously determined optimal conditions, with a graphite dosage of 4 g, and 1 L of reaction liquid. The reaction solution after the exfoliation experiment was diluted 1000 times and then subjected to ultraviolet detection at 660 nm. The concentration of the graphene dispersion was measured to be 3.102 g L^−1^, and the yield of the product was calculated as 3.102 g L^−1^/(4 g/1 L) × 100% = 77.5%.

## 4. Conclusion

In this study, we investigated a new method by combining hydroxyl radical oxidation and electrical exfoliation to produce graphene from graphite. The effect of the exfoliation conditions on the quality of the graphene products was investigated by single factor experiments. The optimal conditions were obtained as 4.0 g of graphite, sodium chloride solution of concentration 5.0% (w/v), applied current strength of 10 mA, and air flow rate of 1.0 L h^−1^ for 3 h. Under optimized conditions, the number of layers of graphene product is about 3, while the products from the individually performed hydroxyl radical oxidation experiment and electrical exfoliation experiment were 50 and 133 layers under the same experimental conditions, respectively. The good quality of the graphene product can be attributed to the synergistic effects of strong oxidation of the hydroxyl radicals and electrical exfoliation. The method has the advantages of simple processing, mild conditions (atmospheric pressure and room temperature), non-utilization of aggressive reagents, recyclability of the reaction medium, *etc.* In general, the new method could be a green and potential method for the production of graphene and graphene derivatives in industry.

## Conflicts of interest

There are no conflicts to declare.

## Supplementary Material
